# Abnormal temporal lobe morphology in asymptomatic relatives of patients with hippocampal sclerosis: A replication study

**DOI:** 10.1111/epi.14575

**Published:** 2018-10-15

**Authors:** Siti Nurbaya Yaakub, Gareth J. Barker, Sarah J. Carr, Eugenio Abela, Michalis Koutroumanidis, Robert D. C. Elwes, Mark P. Richardson

**Affiliations:** ^1^ Department of Basic & Clinical Neuroscience Institute of Psychiatry, Psychology & Neuroscience King’s College London London UK; ^2^ Department of Neuroimaging Institute of Psychiatry, Psychology & Neuroscience King’s College London London UK; ^3^ Department of Clinical Neurophysiology and Epilepsies Guy’s and St. Thomas’ NHS Foundation Trust London UK; ^4^ Department of Clinical Neurophysiology King’s College Hospital London UK

**Keywords:** endophenotype, hippocampal sclerosis, mesial temporal lobe epilepsy, MRI

## Abstract

We investigated gray and white matter morphology in patients with mesial temporal lobe epilepsy with hippocampal sclerosis (mTLE+HS) and first‐degree asymptomatic relatives of patients with mTLE+HS. Using T1‐weighted magnetic resonance imaging (MRI), we sought to replicate previously reported findings of structural surface abnormalities of the anterior temporal lobe in asymptomatic relatives of patients with mTLE+HS in an independent cohort. We performed whole‐brain MRI in 19 patients with mTLE+HS, 14 first‐degree asymptomatic relatives of mTLE+HS patients, and 32 healthy control participants. Structural alterations in patients and relatives compared to controls were assessed using automated hippocampal volumetry and cortical surface–based morphometry. We replicated previously reported cortical surface area contractions in the ipsilateral anterior temporal lobe in both patients and relatives compared to healthy controls, with asymptomatic relatives showing similar but less extensive changes than patients. These findings suggest morphologic abnormality in asymptomatic relatives of mTLE+HS patients, suggesting an inherited brain structure endophenotype.

## 1 INTRODUCTION

Mesial temporal lobe epilepsy with hippocampal sclerosis (mTLE+HS) is conventionally considered to be an acquired disorder, but recent studies indicate that there may be genetic contributions to specific forms of mTLE+HS,^1^, ^2^ suggesting it may be a complex polygenic disorder involving genetic and environmental factors. The study of structural magnetic resonance imaging (MRI) endophenotypes in mTLE+HS may help provide intermediate markers as starting points for genetic analysis.

In sporadic mTLE+HS, the evidence for altered hippocampi in asymptomatic relatives is mixed. Some studies have reported small asymmetric hippocampi in relatives, particularly where there is a strong family history of epilepsy,^3^ whereas other studies have reported no significant hippocampal abnormalities in relatives.^4^, ^5^, ^6^ Only one other study to date has investigated extrahippocampal brain morphology changes in unaffected siblings of patients with sporadic mTLE+HS,^7^ where alterations of cortical morphology were found in anteromedial regions of the ipsilateral temporal lobe in patients; subtle changes were identified in the left entorhinal cortex and parahippocampal gyrus in unaffected siblings of left mTLE+HS probands, and right entorhinal cortex and temporal pole in siblings of right mTLE+HS probands. These changes represented volume loss driven by contractions of cortical surface area in both groups, indicating a possible inherited endophenotype for sporadic mTLE+HS. We regard this evidence of an inherited endophenotype of mTLE+HS as exceptionally important because it suggests a hitherto unsuspected biomarker, with important potential for genetic investigations in this disorder. We believe an adequately powered replication is essential.

The present study aims to characterize volumetric and morphometric structural alterations of gray matter in mTLE+HS patients and asymptomatic relatives, seeking to confirm previous findings, in an independent cohort.

## 2 METHODS

The study was approved by the local research ethics committee, and written informed consent was obtained from each participant.

### 2.1 Participants

Nineteen unrelated patients with unilateral mTLE+HS were recruited from outpatient epilepsy and neurology clinics in hospitals in London, United Kingdom. The diagnosis of mTLE+HS was made on the basis of clinical evaluation including seizure semiology and history, scalp electroencephalography (EEG) including ictal recordings where available, and conventional clinical MRI (details given in Table S1). Patients who had other pathologies, for example, malformations of cortical development or tumors, who had undergone surgical resection of the affected temporal lobe, or who had recent invasive brain investigations (including depth electrode recordings) were excluded from the study.

A group of 14 asymptomatic first‐degree relatives were recruited either through patients in the study or through patients who had a diagnosis of unilateral mTLE+HS but were excluded from the study due to a history of surgical resection or recent invasive brain investigations. Relatives had no current or previous clinical diagnosis of neurologic disorders and a review of history confirmed no events compatible with seizures or complex febrile convulsions (see Table S2). Scalp EEG carried out in all relatives as part of the study showed no epileptiform discharges.

Thirty‐two healthy control participants with no current or previously diagnosed personal or family history of neurologic disorders were recruited for comparison. Demographic information for all participants is shown in Table 1.

**Table 1:** Demographic information and hippocampal volumes for all participants and clinical information for patients in the present study

	Patients	Relatives	Controls
Number	19	14	32
Age (years)	41.3 ± 11.8	31.0 ± 11.9	36.7 ± 10.7
Sex (male/female)	9/10	8/6	15/17
mTLE side^a^ (right/left)	8/11	7/7	
Epilepsy onset age (years)	23.6 ± 11.6	–	–
Duration of epilepsy (years)	17.3 ± 13.8	–	–
Hippocampal volume^b^ (cm^3^)			
Ipsilateral	3.53 ± 0.57	4.51 ± 0.58	4.52 ± 0.43
Contralateral	4.47 ± 0.50	4.66 ± 0.41	4.53 ± 0.45

Data are means ± standard deviations unless stated otherwise.

a Refers to side of seizure onset in patient group, and of the proband in relative group.

b Volumes are corrected for total intracranial volume. Ipsilateral and contralateral are relative to the pathologic hippocampus in the patient or proband after side‐flipping.

### 2.2 MRI acquisition

MRI data collection was performed on a General Electric 3T MR750 scanner (GE Healthcare Systems, Chicago, Illinois) using the body coil for radiofrequency transmission and a 12‐channel head coil for signal reception. A three‐dimensional inversion recovery–prepared spoiled gradient‐echo image was acquired in the sagittal plane with a 270 mm field of view, 256 × 256 matrix (voxel size: 1.05 × 1.05 mm), 196 slices of 1.2 mm thickness, 7 msec repetition time, 400 msec inversion time, 3 msec echo time, and 11‐degree excitation flip angle.

### 2.3 Data processing

To increase the power to detect pathologic differences, imaging data from patients with right‐sided HS (n = 8; 42.1%) and relatives of patients with right‐sided HS (n = 7; 50.0%) were side‐flipped so that the ipsilateral side is on the left. All changes were considered as ipsilateral or contralateral to the pathologic hippocampus. For consistency, a proportion of healthy control data (n = 15; 46.9%) was chosen randomly to be side‐flipped.

MRI images were analyzed using automatic hippocampal volumetry, to identify overall hippocampal volume abnormalities, and surface‐based morphometry (SBM) for analysis of cortical surface morphology. Additional exploratory analyses of voxel‐wise gray and white matter morphometry are reported in Appendix S1.

MRI images were first processed using the automated *recon‐all* FreeSurfer processing stream (version 5.3.0; http://surfer.nmr.mgh.harvard.edu), which has been validated and documented elsewhere.^8^, ^9^ Quality checks were performed according to the ENIGMA consortium pipeline (http://enigma.usc.edu).^10^

### 2.4 Hippocampal volumetry

Following FreeSurfer processing, hippocampal volumes and total intracranial volumes (ICVs) were obtained in native space from FreeSurfer. To account for interindividual differences in brain volume, hippocampal volumes were separately adjusted for total ICV using a covariance model^3^

$${\mathrm{HV}}_{\mathrm{corrected}}={\mathrm{HV}}_{\mathrm{measured}}-\mathrm{gradient}\times \left({\mathrm{ICV}}_{\mathrm{measured}}-{\mathrm{ICV}}_{\mathrm{mean}}\right)$$

where the gradient is the linear regression fit of hippocampal volume to ICV in the control group, and ICV_mean_ is the average ICV of controls. Group differences in the corrected hippocampal volumes were assessed using Student’s *t‐*tests.

### 2.5 Surface‐based morphometry

Vertex‐wise cortical surface morphology was assessed using volume, surface area, and cortical thickness measures at each vertex within FreeSurfer’s *QDEC* functionality. Subjects were mapped to an average template subject and smoothed using a 10 mm full‐width at half maximum Gaussian. Group differences in surface morphology were assessed using a general linear model applied at each vertex with gender and age as covariates. Because surface area and brain volumes have been shown to vary with head size,^11^ ICV was additionally included as a covariate. Corrections for multiple comparisons were performed using Monte Carlo simulations with a two‐tailed clusterwise threshold of *P *<* *0.05, adjusted for 6 multiple comparisons (3 measures and 2 hemispheres) using Bonferroni correction (i.e., clusters were significant at *P *<* *0.008).

## 3 RESULTS

### 3.1 Hippocampal volumes

Hippocampal volumes are summarized in Table 1. Compared with controls, patients had significantly smaller ipsilateral hippocampal volumes; *t*(49) = −7.02, *P *<* *0.001. Contralateral hippocampal volumes were not significantly different between patients and controls, and there were no significant differences between relatives and controls.

### 3.2 Surface‐based morphometry

**Figure 1:**
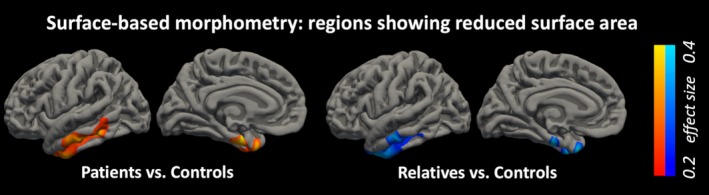
Surface‐based morphometry results. Clusters showing reduced cortical surface area in patients (red/yellow) and relatives (blue/cyan) compared to controls in the ipsilateral temporal lobe (clusters survive cluster correction at *P* < 0.05 and Bonferroni correction for 6 comparisons)

Only cortical surface area showed significant group differences. Patients showed a surface area that was significantly reduced relative to that of controls in the ipsilateral temporal lobe extending over the middle and inferior temporal gyri, temporal pole, fusiform gyrus, and entorhinal cortex (*P *=* *0.004). Relatives also showed significantly reduced surface area in the ipsilateral temporal lobe (*P = *0.002); this region had substantial overlap with, but a smaller spatial extent than, the region found abnormal in the patients (Figure 1).

## 4 DISCUSSION

We characterized volumetric and morphometric structural abnormalities in patients with mTLE+HS and asymptomatic relatives using T1‐weighted structural MRI. SBM demonstrated a shared reduction in ipsilateral temporal lobe cortical surface area, primarily in the inferior temporal gyrus, fusiform, and entorhinal cortex, in both mTLE+HS patients and relatives compared to healthy controls. The surface area reduction in relatives was unilateral and in the same hemisphere as seizure onset in the proband. This represents the first replication of an ipsilateral surface‐based morphologic endophenotype in asymptomatic relatives of mTLE+HS patients.

The finding of hippocampal atrophy is expected in this cohort of patients with mTLE+HS. Although it is widely accepted that structural abnormalities in patients with mTLE+HS extend beyond the epileptogenic hippocampus,^12^ it is unclear whether these changes result from damage due to recurrent seizures,^13^ represent an inherent predisposition to epilepsy, or are a combination of the 2 effects.^14^ Furthermore, some studies have reported that the severity of morphologic abnormality correlates with epilepsy duration and age at onset, whereas others have found no clear relationship.^12^

Only one other study has reported altered cortical surface morphology in asymptomatic relatives of patients with sporadic mTLE+HS,^7^ in the same region of temporal cortex and predominantly on the same side as HS in probands. The study presented here is strong supportive evidence of a temporal cortex surface area endophenotype in mTLE+HS, in an independent dataset. Of interest, ipsilateral findings in both studies may suggest a predisposition toward side of HS. In addition, in exploratory analyses reported in Appendix S1, we found evidence, albeit weaker, of unilateral hippocampal volume loss in relatives and bilateral white matter volume loss in the underlying white matter of the temporal lobes in relatives. Although these weaker effects in relatives did not reach our threshold for significance, in the context of other significant findings in relatives, we argue that there is a compelling emerging picture of a structural endophenotype in relatives of patients with mTLE+HS.

Structural brain abnormality in extrahippocampal regions in relatives of patients with mTLE+HS is important evidence that extrahippocampal structural abnormality in patients cannot be entirely a consequence of seizures and epilepsy. Although there is accumulating evidence for genetic susceptibility to mTLE+HS, related to variation in the *SCN1A* sodium channel gene or to the genetic control of pro‐ictogenic inflammatory pathways,^1^, ^2^ the underlying genetic cause of the structural imaging endophenotype reported here remains unknown. One component of the endophenotype, cortical surface area change in anterior temporal cortex, has now been identified in 2 independent datasets, justifying future genetic investigation of this feature.

There is good evidence that anterior temporal cortex is critically involved in seizure onset in mTLE+HS,^15^ challenging the concept that seizures in mTLE+HS are driven entirely by the hippocampus. Our finding here of a structural imaging endophenotype affecting this region in relatives suggests an underlying potentially ictogenic abnormality, which may be related to the observation of ictal onset in this area in some cases of mTLE+HS.

The present study is limited by the small sample size of the groups, which did not allow us to consider left and right mTLE patients separately. From previously reported effect sizes, however,^7^ we should be able to detect cortical surface morphology alterations with 16 patients and 14 relatives with 80% power, indicating that the study is sufficiently powered for this replication analysis.

Although our primary motivation was to replicate the prior study of Alhusaini et al,^7^ our study has some methodologic differences. Our study was carried out using a 3T scanner, whereas the prior study was at 1.5T. We selected relatives who were predominantly unrelated to the patients in the study, whereas Alhusaini and colleagues studied patients and same‐sex siblings. We argue that this methodologic difference further reinforces the point that there is a common structural endophenotype despite potential genetic heterogeneity. Alhusaini et al^7^ identified a region of temporal cortex with different surface morphology in the comparison between patients and healthy controls, using a highly sensitive compound measure referred to as “metric distortion,” and subsequently compared cortical surface area, volume, and thickness between relatives and healthy controls in that region. In our analysis, we compared surface morphology measures between relatives and controls across the whole brain rather than only from a region found to be significantly different at the whole brain level between patients and controls. Our more conventional and direct whole‐brain analysis approach is more suited to a replication, to confirm and further localize these differences between relatives and controls. Otherwise, our analysis was extremely similar to that of the prior study. Despite these methodologic and analytic differences, we were still able to replicate the results reported by Alhusaini and colleagues, providing further evidence for a shared structural endophenotype despite potential genetic heterogeneity.

Studies of psychiatric disorders have identified several candidate endophenotypes, but none have yet been successful at identifying specific susceptibility genes for psychiatric disorders,^16^ which may be because the genetic associations in psychiatric disorders are less clear. The foundations for genetic associations in epilepsy are much firmer, and endophenotypes have been proven to help in the search for susceptibility genes in Rolandic epilepsy.^17^, ^18^ However, many individual studies lack the statistical power to identify risk genes due to small sample sizes. Initiatives such as the Enhancing Neuro Imaging Genetics through Meta‐Analysis project for epilepsy (ENIGMA; http://enigma.loni.ucla.edu/) have been working to consolidate data from multiple groups and collaboratively investigate new genetic risk variants associated with epilepsy.

## 5 CONCLUSION

Our finding of structural alterations in asymptomatic relatives of patients with mTLE+HS represents strong evidence for an inherited endophenotype in brain morphology. This endophenotype may represent a genetic predisposition to mTLE that is manifest as epilepsy only if there is additional hippocampal injury perhaps from an independent environmental exposure.

## DISCLOSURE OF CONFLICT OF INTEREST

Gareth J. Barker received honoraria for teaching from General Electric Healthcare during the course of this study, from whom he also receives grants funding for a PhD student, and acts as a consultant for IXICO. All other authors report no disclosures. We confirm that we have read the Journal’s position on issues involved in ethical publication and affirm that this report is consistent with those guidelines.
