# Supplementary material for: Abnormal temporal lobe morphology in asymptomatic relatives of patients with hippocampal sclerosis: A replication study
Source: Epilepsia. 2018 Oct 15;60(1):e1–5. doi: 10.1111/epi.14575 (PMC6334277; doi:10.1111/epi.14575)
Supplement: Supplementary file 1 [file EPI-60-e1-s001.docx]

**Supplementary Table 1.** Further clinical details of patients in the study.

| **ID** | **Age** | **Sex** | **Onset age** | **MRI and ictal EEG features** | **Seizure frequency (per month)** | **Medication (daily dose, mg)** | **History of febrile convulsions?** |
| --- | --- | --- | --- | --- | --- | --- | --- |
| PAT01 | 41 | F | 24 | Right HS on MRI. Ictal EEG not obtained. | 9-10 | LMT (550); LEV (1500); PER (4); CLB (60) | Unknown |
| PAT02 | 35 | M | 15 | Left HS on MRI. Ictal EEG not obtained. | 2 | LMT (400); VPA (900); CLB (10) | Unknown |
| PAT03 | 43 | F | 20 | Left HS on MRI. Independent left and right temporal ictal onsets (intracranial EEG). | 3-7 | CBZ (1400); LMT (200) | None |
| PAT04 | 57 | F | 5 | Left HS on MRI. Left temporal interictal discharges; lateralization of ictal onset unclear (scalp EEG). | 6-7 | LEV (875); CIT (30) | None |
| PAT05 | 22 | M | 16 | Left HS on MRI. Left temporal ictal onset (scalp EEG). | 20-24 | CBZ (1200) | None |
| PAT06 | 34 | M | 11 | Right HS on MRI. Right temporal ictal onset (scalp EEG). | 2-3 | PHB (60); VPA (1200); OLA (7.5); CIT(*) | None |
| PAT07 | 51 | F | 31 | Left HS on MRI. Left temporal interictal discharges; right temporal ictal onset (scalp EEG). | <1 | LAC (400) | Unknown |
| PAT08 | 31 | M | 25 | Right HS on MRI. Ictal EEG not obtained. | 16-20 | CBZ (800); LEV (200); CLB (10) | Unknown |
| PAT09 | 48 | M | 33 | Right HS on MRI. Ictal EEG not obtained. | <1 | LEV (3000); TOP (100) | Yes |
| PAT10 | 58 | F | 47 | Left HS on MRI. Ictal EEG not obtained. | 3-4 | TOP (200); CLB (10) | Unknown |
| PAT11 | 47 | M | 40 | Right HS on MRI. Ictal EEG not obtained. | <1 | LEV (400) | Unknown |
| PAT12 | 43 | F | 40 | Left HS on MRI. Ictal EEG not obtained. | 4 | CBZ (800) | None |
| PAT13 | 23 | M | 22 | Right HS on MRI. Ictal EEG not obtained. | <1 | ZON (200) | Yes |
| PAT14 | 57 | M | 25 | Right HS on MRI. Ictal EEG not obtained. | <1 | LMT (300) | Unknown |
| PAT15 | 37 | F | 27 | Left HS on MRI. Ictal EEG not obtained. | 12 | CBZ (400) | Yes |
| PAT16 | 31 | F | 22 | Left HS on MRI. Ictal EEG not obtained. | 3 | LEV (3000) | None |
| PAT17 | 44 | F | 1 | Left HS on MRI. Ictal EEG not obtained. | 4 | CBZ (1200); CLB (10) | Unknown |
| PAT18 | 22 | F | 18 | Left HS on MRI. Ictal EEG not obtained. | 1-2 | None at time of scan | None |
| PAT19 | 52 | M | 25 | Right HS on MRI. Ictal EEG not obtained. | 15 | LMT (750); PER (8) | Unknown |

HS = Hippocampal Sclerosis; FS = Febrile Seizures. * = unknown dose

Drug abbreviations: CBZ = Carbamazepine, CIT = Citalopram, CLB = Clobazam, LAC = Lacosamide, LEV = Levetiracetam, LMT = Lamotrigine, OLA = Olanzapine, PER = Perampanel, PHB = Phenobarbitone, TOP = Toparimate, VPA = Valproate, ZON = Zonisamide.

**Supplementary Table 2.** Further details of relatives and clinical details of associated probands.

| **ID** | **Age** | **Sex** | **History of febrile convulsions?** | **Relationship to proband** | **Proband mTLE details** | **Proband in study** |
| --- | --- | --- | --- | --- | --- | --- |
| REL01 | 49 | M | None | Son of female patient | Left HS on MRI. History of febrile convulsions unknown. Patient has not undergone surgery. | No |
| REL02 | 34 | M | Unknown | Twin brother of male patient | Right HS on MRI. No history of febrile convulsions. Patient has not undergone surgery. | PAT06 |
| REL03 | 37 | F | Unknown | Daughter of female patient | Left HS on MRI. No history of febrile convulsions. Patient has not undergone surgery. | PAT04 |
| REL04 | 31 | F | None | Daughter of male patient | Right HS on MRI. No history of febrile convulsions. Patient has undergone right mesial temporal resection. Pathology confirmed mesial temporal sclerosis. | No |
| REL05 | 17 | M | None | Son of female patient | Left HS on MRI. No history of febrile convulsions. Patient has undergone left mesial temporal resection. Pathology confirmed mesial temporal sclerosis. | No |
| REL06 | 25 | M | One simple FS aged 2. | Brother of female patient | Right HS on MRI. No history of febrile convulsions. Patient has undergone right mesial temporal resection. Pathology confirmed mesial temporal sclerosis. | No |
| REL07 | 30 | M | None | Son of female patient | Left HS on MRI. History of febrile convulsions unknown. Patient has undergone left mesial temporal resection. Pathology confirmed mesial temporal sclerosis. | No |
| REL08 | 25 | M | None | Brother of male patient | Right HS on MRI. Positive history of febrile convulsions. Patient has not undergone surgery. | PAT13 |
| REL09 | 24 | F | None | Daughter of female patient | Right HS on MRI. No history of febrile convulsions. Patient has not undergone surgery. | No |
| REL10 | 20 | F | None | Daughter of female patient | Left HS on MRI. History of febrile convulsions unknown. Patient has undergone left mesial temporal resection. Pathology confirmed mesial temporal sclerosis. | No |
| REL11 | 60 | F | None | Mother of female patient | Left HS on MRI. Prolonged febrile convulsions at 8 months. Patient has undergone left mesial temporal resection. Pathology confirmed mesial temporal sclerosis. | No |
| REL12 | 28 | M | Two simple FS aged 18 months & 2 years. | Brother of female patient | Left HS on MRI. Prolonged febrile convulsions at 8 months. Patient has undergone left mesial temporal resection. Pathology confirmed mesial temporal sclerosis. | No |
| REL13 | 29 | F | None | Daughter of female patient | Right HS on MRI. History of febrile convulsions unknown. Patient has not undergone surgery. | No |
| REL14 | 17 | M | None | Son of female patient | Right HS on MRI. History of febrile convulsions unknown. Patient has not undergone surgery. | No |

**Supplementary Material: Voxel-Based Morphometry Analysis**

**Method**

Voxel-wise alterations in grey and white matter volumes were separately investigated using VBM implemented in SPM12 (http://www.fil.ion.ucl.ac.uk/spm).^1^ Briefly, this involved tissue segmentation, *DARTEL* for iteratively estimating deformations to align each subject’s grey and white matter segments to a custom average template,^2^ spatial normalisation to the MNI standard space atlas, modulation with ICV, and spatial smoothing with a 10mm full-width at half maximum Gaussian. Group differences in grey or white matter volumes were assessed using a general linear model (GLM) applied at each voxel with age, gender and ICV as covariates with group differences identified using permutation testing and voxel-wise correction within the grey or white matter mask (implemented with FSL’s *randomise*^3^).

**Results**

Using VBM (Supplementary Figure 1A&B), patients showed reduced grey matter volume in the ipsilateral hippocampus and reduced volume in the white matter in the ipsilateral inferior temporal gyrus. There were no significant volume differences between controls and relatives. At the uncorrected level of *p*<0.01, there was evidence for grey matter volume reduction in the ipsilateral hippocampal tail, and white matter volume reduction around the posterior middle temporal and fusiform gyri bilaterally in relatives compared to controls.

**Discussion**

Using VBM, significant atrophy was found in the ipsilateral hippocampus of mTLE+HS patients, but only weak evidence for atrophy in the hippocampal tail in relatives. There was also weak evidence for abnormal white matter in the bilateral temporal lobes in relatives.


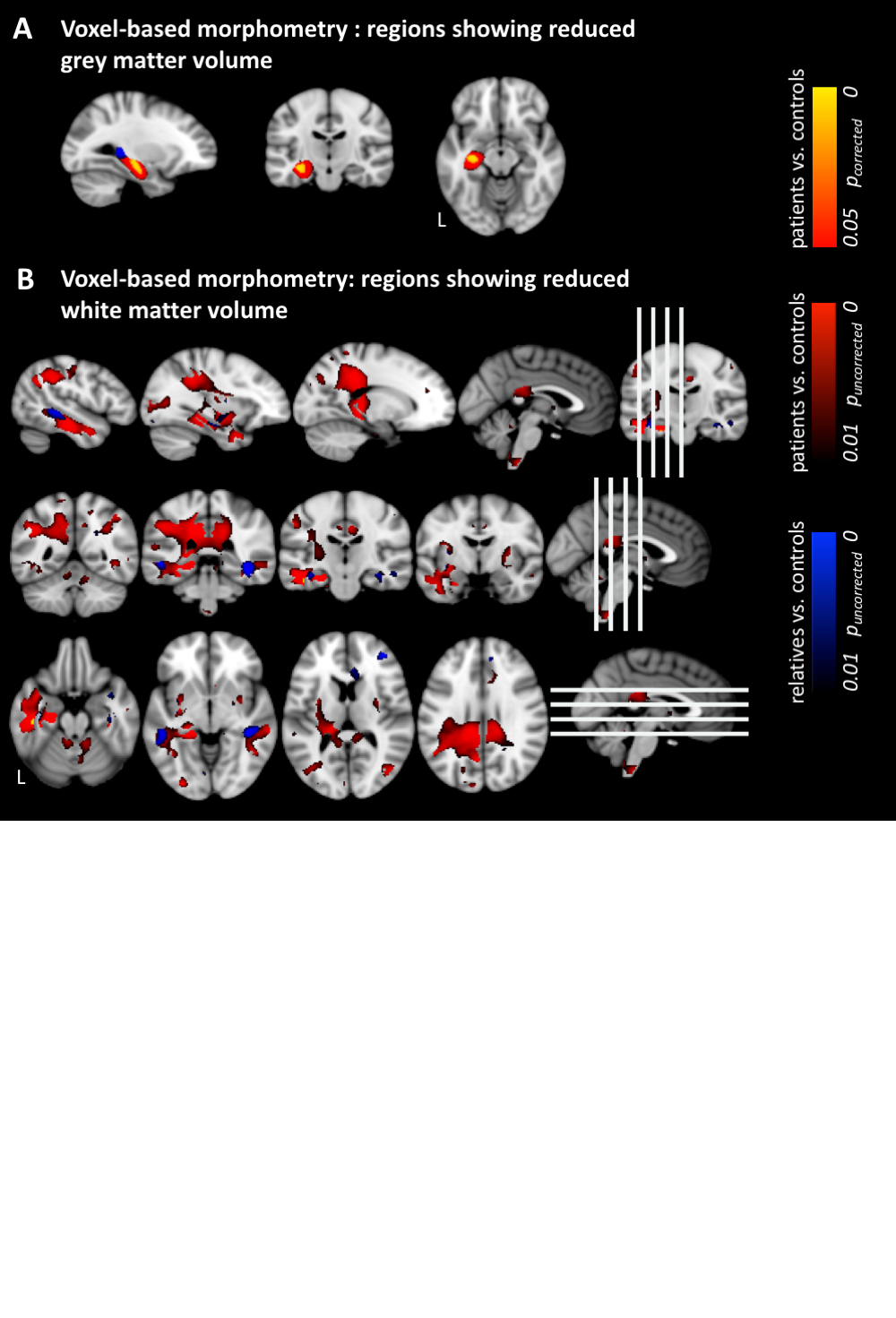


**Supplementary Figure 1. Voxel- and surface-based morphometry results.** Regions showing reduced **(A)** grey matter volume and **(B)** white matter volume in mTLE patients compared to controls (in red/yellow), and in asymptomatic relatives compared to healthy controls (in blue) from voxel-based morphometry analysis. Clusters in red/yellow show statistically significant grey matter reduction in mTLE patients compared to controls in the ipsilateral hippocampus, shown here after voxel-wise correction at p<0.05. Clusters where reduced GMV in did not reach statistical significance (p<0.01, uncorrected) are shown in dark red for patients vs. controls, and in dark blue for relatives vs. controls. L indicates ipsilateral side and the x, y, and z coordinates in MNI space are shown above each slice.

**References**

1. Ashburner J, Friston KJ. Voxel-Based Morphometry - The Methods. Neuroimage 2000; 11: 805–821.

2. Ashburner J. A fast diffeomorphic image registration algorithm. Neuroimage 2007; 38: 95–113.

3. Winkler AM, Ridgway GR, Webster MA, Smith WN, Nichols TE. Permutation inference for the general linear model. Neuroimage 2014; 92: 381-397.
